# Identification of Novel Risk Variants of Non-Syndromic Cleft Palate by Targeted Gene Panel Sequencing

**DOI:** 10.3390/jcm12052051

**Published:** 2023-03-04

**Authors:** Justyna Dąbrowska, Barbara Biedziak, Agnieszka Bogdanowicz, Adrianna Mostowska

**Affiliations:** 1 Department of Biochemistry and Molecular Biology, Poznan University of Medical Sciences, 6 Swiecickiego Street, 60-781 Poznan, Poland; 2 Department of Orthodontics and Craniofacial Anomalies, Poznan University of Medical Sciences, 60-812 Poznan, Poland

**Keywords:** non-syndromic cleft palate, rare coding variants, pathogenic variants, NGS gene panel

## Abstract

Non-syndromic cleft palate (ns-CP) has a genetically heterogeneous aetiology. Numerous studies have suggested a crucial role of rare coding variants in characterizing the unrevealed component of genetic variation in ns-CP called the “missing heritability”. Therefore, this study aimed to detect low-frequency variants that are implicated in ns-CP aetiology in the Polish population. For this purpose, coding regions of 423 genes associated with orofacial cleft anomalies and/or involved with facial development were screened in 38 ns-CP patients using the next-generation sequencing technology. After multistage selection and prioritisation, eight novel and four known rare variants that may influence an individual’s risk of ns-CP were identified. Among detected alternations, seven were located in novel candidate genes for ns-CP, including *COL17A1 (*c.2435-1G>A), *DLG1* (c.1586G>C, p.Glu562Asp), *NHS (*c.568G>C, p.Val190Leu—de novo variant), *NOTCH2* (c.1997A>G, p.Tyr666Cys), *TBX18* (c.647A>T, p.His225Leu), *VAX1* (c.400G>A, p.Ala134Thr) and *WNT5B* (c.716G>T, p.Arg239Leu). The remaining risk variants were identified within genes previously linked to ns-CP, confirming their contribution to this anomaly. This list included *ARHGAP29* (c.1706G>A, p.Arg569Gln), *FLNB* (c.3605A>G, Tyr1202Cys), *IRF6* (224A>G, p.Asp75Gly—de novo variant), *LRP6* (c.481C>A, p.Pro161Thr) and *TP63* (c.353A>T, p.Asn118Ile). In summary, this study provides further insights into the genetic components contributing to ns-CP aetiology and identifies novel susceptibility genes for this craniofacial anomaly.

## 1. Introduction

Orofacial clefts (OFCs) are one of the most common congenital malformations, affecting 1 in 700 live-born children worldwide [1]. Treatment of those developmental anomalies requires long-term, multi-specialist care, including craniofacial and dental corrective surgeries, speech and hearing therapy, and psychosocial support [2,3]. Moreover, the occurrence of OFCs is frequently associated with an increased risk of various cancers [2,4].

OFCs are divided into two primary forms: cleft lip with or without cleft palate (CL/P) and cleft palate (CP). Interestingly, the embryological and epidemiological data suggest a distinct developmental mechanism and genetic aetiology of these two subtypes [5,6,7,8,9]. Globally, CP occurs in 6 per 10,000 live births [10]. Approximately 50% of all CP cases are non-syndromic (ns-CP) [8], appearing in isolation with no other structural or cognitive anomalies and are aetiologically different from those occurring with cleft syndromes [9].

To date, multiple analyses have established the crucial role of genetic components in the complex aetiology of ns-CP [9,11,12], but the genetic basis of ns-CP has not been fully elucidated. The encountered issues are related to the considerable contribution of environmental factors to the ns-CP aetiology and the diversity in susceptibility loci and variants depending on the racial background, which suggests that the genetic architecture of ns-CP may vary between different populations [11,13,14,15].

Numerous studies, including genome-wide association studies (GWASs) and meta-analyses conducted in different populations, identified only several significant risk loci for ns-CP [15,16,17,18,19]. The strongest signal within detected chromosomal regions was located in the *GRHL3* gene (OMIM * 608317), encoding a Grainyhead-like transcriptional factor that stimulates the migration of endothelial cells during embryonic palate development. The most significant *GRHL3* variant that reached the genome-wide significance level was a deleterious transition c.1361C>T, leading to a threonine to methionine substitution at position 454 (rs41268753) [16]. The pathogenic variants of *GRHL3* have also been reported to cause Van der Woude syndrome (OMIM #119300), the most common disorder associated with orofacial clefts [20]. Interestingly, the GWASs revealed that the top variants that reached the genome-wide significance threshold had a relatively low frequency in the studied populations [14,21], suggesting that rare functional nucleotide alternations might be more relevant in understanding the molecular background of ns-CP than common nucleotide changes [22,23]. Therefore, several previous studies applied next-generation sequencing (NGS) to identify rare coding variants that may be involved in the aetiology of this structural anomaly. The deleterious alternations were mainly detected within known candidate genes and genes highly expressed in mouse facial primordia [24,25,26,27,28,29].

Therefore, this study aimed to identify low-frequency functional variants that may contribute to ns-CP aetiology. For this purpose, the coding regions of 423 genes associated with orofacial clefts and/or encoding proteins involved in facial development were analysed using the NGS-based multi-gene panel in 38 ns-CP patients from the Polish population.

## 2. Materials and Methods

### 2.1. Study Population

The participants were recruited from the genetically homogenous Polish population through The Comprehensive Therapy Program for Craniofacial Anomalies at Poznan University of Medical Sciences. The study consisted of 38 ns-CP patients with no other structural or cognitive anomalies, of which, in 8 ns-CP cases (21.05%), dental anomalies were reported (Table 1). However, since these are expected consequences associated with cleft palate, they were not excluded from the analysis. A total of 35 recruited patients (92.11%) had a cleft of the hard and soft palate, while, in 3 cases (7.89%), the cleft affected only the soft palate. Only 4 participants (10.53%) had a family history of ns-CP. Peripheral blood samples from ns-CP patients and their parents, that is, 34 complete triads (89.47%) and 4 dyads (10.52%), were collected to isolate genomic DNA by salt extraction. This study was conducted according to the Declaration of Helsinki [30] and was approved by the Ethics Committee of the Poznan University of Medical Science (no 1115/18). All participants or their legal guardians provided written informed consent.

### 2.2. The NGS-Based Multi-Gene Panel

The whole coding sequence and flanking regions [exon-intron junction (+/−55 bp)] of 423 genes were tested to identify rare risk variants associated with ns-CP. The multi-gene panel was previously designed to detect nucleotide changes involved in the aetiology of orofacial dysmorphologies, including non-syndromic cleft lip with or without cleft palate (ns-CL/P) and non-syndromic tooth agenesis (ns-TA); please see our previous studies [31,32] for the detailed methodology and strategy for selecting the genes. In brief, the panel consisted of genes with previously documented association with ns-OFCs, those linked to cleft syndromes and/or dental anomalies, for which knockout mice models display OFCs and/or dental anomalies, are highly expressed in craniofacial regions during embryonic development, encode proteins engaged in signalling pathways during facial development and those involved in DNA repair mechanisms. The characteristics of 423 genes analysed are presented in Tables S1 and S2. Based on the provided chromosomal coordinates, the set of DNA probes complementary to the target regions (SeqCap^®^ EZ Prime Choice, Roche Sequencing Solutions, CA, USA) was designed using the NimbleDesign^®^ online tool (Roche, WI, USA). The cumulative length of the chosen regions was 1.59 Mb with a 98.5% coverage rate. The KAPA HyperPlus and KAPA Library Quantification kits (Roche Sequencing Solutions, CA, USA) were used to prepare the sequencing libraries and quantification of the prepared DNA samples, respectively. The targeted and enriched exonic regions were sequenced with the 150 bp paired-end reads using the HiSeq 4000 System (Ilumina Inc., San Diego, CA, USA) according to the manufacturer’s protocols.

#### 2.2.1. Variant Identification and Annotation

The detailed methodology, including sequencing, data analysis and quality control steps, was performed as previously described [31,32]. Briefly, the sequencing reads that passed quality control were aligned using Burrows–Wheeler Aligner software (BWA; http://bio-bwa.sourceforge.net (accessed on 1 March 2022)) to the UCSC assembly GRChC38/hg38 (https://genome.ucsc.edu (accessed on 1 March 2022)) as a human genome reference sequence. The Genome Analysis Toolkit (GATK, http://www.broadinstitute.org/gatk/ (accessed on 1 March 2022)) was used to call single nucleotide variants and short insertions/deletions. Subsequently, the identified variants were annotated with the databases to provide information regarding their frequency (gnomAD, https://gnomad.broadinstitute.org (accessed on 1 March 2022)) and clinical significance (HGMD, http://www.hgmd.cf.ac.uk (accessed on 1 March 2022) and ClinVar, https://www.ncbi.nlm.nih.gov/clinvar (accessed on 1 March 2022)). Pathogenicity predictions for the variants were uploaded using the genomic variant search engine VarSome (https://varsome.com (accessed on 1 March 2022)), which provided verdicts from 17 in silico tools (SIFT, DEOGEN2, EIGEN, EIGEN PC, FATHMM, FATHMM—MKL, FATHMM—XF, LRT, Meta LR, Meta SVM, Mutation Assessor, Mutation Taster, MVP, PrimateAI, PROVEAN, REVEL, SHIT4G). Additionally, the Combined Annotation Dependent Depletion (CADD) (https://cadd.gs.washington.edu/snv (accessed on 1 March 2022)) scores and adaptive boosting (ADA) scores were provided, respectively, for the missense and splicing variants.

#### 2.2.2. Variant Selection and Prioritisation

The analysis was limited to exonic and splicing variants with a minor allele frequency (MAF) < 0.001 in the gnomAD European (non-Finnish) population. To select variants that may substantially impact encoded protein functions or the splicing process, stop-gain, stop-loss, and frameshift variants, as well as variants with an ADA score ≥ 0.9 and missense variants with a CADD score ≥ 20 were considered. In addition, their pathogenicity must have been confirmed by at least half of the employed in silico tools. Variants detected within genes with the documented association with non-syndromic or syndromic OFCs and genes encoding proteins playing an essential role in craniofacial development were prioritised. Moreover, the selected alternations were ranked by their segregation patterns and classified into three categories: de novo, inherited from unaffected parents and unknown inheritance.

#### 2.2.3. Variant Validation and Segregation

All selected variants were confirmed by direct Sanger sequencing, and segregation analyses were conducted in the case-parents trios when DNA samples were available. The ABI Prism™ BigDye™ Terminator Cycle Sequencing kit was employed using the ABI Prism 3730 capillary sequencer (Applied Biosystems, Foster City, CA, USA) to sequence the PCR products.

## 3. Results

### Targeted NGS Sequencing

After filtering, prioritisation, confirmation and segregation analyses, twelve risk variants that might have the most pathogenic impact on ns-CP in the study cohort were identified (Supplementary Figure S1). The detailed characteristics, including segregation analysis and pathogenicity predictions for all nucleotide alternations detected in ns-CP patients, are presented in Table 2 and Table 3 and Supplementary Figures S2–S13.

Most of the identified alternations (n = 8) were new changes that had not been previously described, of which two were de novo, including the missense variant p.Asp75Gly (c.224A>G) in IRF6 (OMIM * 607199, pLi = 1.0, Z score = 2.7). This p.Asp75Gly substitution was predicted as pathogenic by 16 out of 17 in silico tools and obtained the CADD score of 28.5. The second de novo missense variant was detected in the NHS gene (OMIM * 300457, pLi = 1.0, Z score = 1.8), which had not been previously linked with ns-OFCs. The NHS c.568G>C transversion leading to a p.Val190Leu substitution was evaluated as disrupting by 7 out of 15 in silico tools and had a CADD score of 24.6.

A total of 5 newly identified risk variants were inherited from an unaffected parent, including the splicing variant c.2435-1G>A in COL17A1 (OMIM * 113811, pLi = 0.0, Z score = 0.7). The ADA score for this variant was 0.99, and the CADD score was 33. The other 4 novel alternations inherited from healthy parents were missense variants detected in genes with positive Z scores ranging from 0.8 to 3.5, including c.3605A>G (p.Tyr1202Cys) in FLNB (OMIM * 603381 pLi = 0.0), c.1997A>G (p.Tyr666Cys) in NOTCH2 (OMIM * 600275, pLi = 1.0), c.353A>T (p.Asn118Ile) in TP63 (OMIM * 603273, pLi = 0.9) and c.400G>A (p.Ala134Thr) in VAX1 (OMIM * 604294, pLi = 0.7). Most of the 17 in silico tools used for pathogenicity prediction (mean 15) confirmed the pathogenic status of these nucleotide changes with CADD scores ranging from 26.1 to 29.2.

The newly identified missense variants included c.647A>T (p.His225Leu) in TBX18 (OMIM * 604613, pLi = 0.7, Z score = 0.8), which has an unknown inheritance due to the lack of the father’s DNA. All 17 bioinformatics programmes applied for in silico analysis predicted this variant as pathogenic, and its CADD score was equal to 31.0.

The remaining 4 identified changes were rare missense variants with allele frequency in the gnomAD European population ranging from 0.00 to 2.21E-05, including c.1706G>A (p.Arg569Gln) in ARHGAP29 (OMIM * 610496), c.1587G>C (p.Glu562Asp) in DLG1 (OMIM * 601014), c.481C>A (p.Pro161Thr) in LRP6 (OMIM * 603507) and c.716G>T (p.Arg239Leu) in WNT5B (OMIM * 606361). Except for p.Arg569Gln in ARHGAP29, which inheritance was unknown due to the lack of the father’s DNA, all these rare variants were inherited from an unaffected parent. The selected risk alternations obtained the pathogenic status from all or most of the applied bioinformatics programmes (from 10 to 17) and had CADD scores ranging from 23.6 to 32.0.

Additional characteristics of nucleotide alternations detected in ns-CP patients are presented in Table S3. Table S4 presents characteristics of likely pathogenic variants not fulfilling the project’s strict selection criteria, which were identified in ns-CP patients.

## 4. Discussion

The aetiology of non-syndromic orofacial clefts consists of multiple genetic components. Since GWASs captured only about 20% of the heritability of nsOFCs [23], many studies have been applied to identify this unrevealed portion of genetic variation and support the essential role of rare functional variants in explaining the “missing heritability” of the ns-OFCs [22,23].

Therefore, this study aimed to detect rare coding variants that are implicated in ns-CP aetiology in the Polish population. For this purpose, coding regions of 423 genes associated with orofacial cleft anomalies and/or involved with facial development were screened in 38 ns-CP patients to identify the top 12 variants that exhibit the most deleterious features. The majority of these alternations were identified in unaffected family members. However, this might provide further support for the heterogeneous nature of the ns-CP [33,34]. The incomplete penetrance of detected pathogenic alternations suggests that they require interactions with other genetics and/or environmental factors to trigger the expression of the cleft phenotype. Therefore, in many studies focusing on the genetics of complex traits and diseases, pathogenic alterations found in asymptomatic carriers are not ruled out from their contribution to disease. Thus, nucleotide variants identified in the current study may be considered one of the genetic components influencing the individual’s risk of ns-CP.

Among selected nucleotide changes, eight were novel and not previously described in the literature. A splicing variant c.2435-1G>A identified in *COL17A1* is particularly noteworthy. The *COL17A1* gene encodes type XVII collagen, which performs the structural function of hemidesmosomes responsible for maintaining the proper adhesion of epithelial cells to the basement membrane [35]. Interestingly, the gene ontology analysis showed that genes encoding collagen-α chain proteins are significantly enriched exclusively in cleft palate phenotypes [36]. The *COL17A1* gene has not been previously linked with ns-CP. However, a GWAS conducted in the European population suggested that nucleotide variants located in *COL17A1* may impact facial morphology [37]. In addition, *COL17A1* expression increased during the growth and fusion of facial prominences in murine models [38]. Additionally, a rare, likely pathogenic variant of *COL17A1* has been described in ns-CLP patients, suggesting that *COL17A1* is a novel candidate for isolated orofacial clefts [29,31].

Other novel susceptibility genes for ns-CP detected in our study included *DLG1*, *NHS*, *NOTCH2*, *TBX18*, *VAX1* and *WNT5B*. While common and rare variants of *DLG1*, *NOTCH2*, *VAX1* and *WNT5B* have been previously linked with the risk of ns-CLP [29,31,39,40,41,42], the *NHS* deleterious variants have been implicated only in Nance–Horan syndrome (OMIM #302350) characterised by craniofacial dysmorphologies [43,44]. The *NHS* gene encodes three isoforms (A–C), and isoform A is highly expressed during embryonic development in the mesenchymal tissue within the craniofacial and tooth buds regions and plays an essential role in remodelling the actin filaments and maintaining cell morphology [43,44,45]. The *NHS* variant (p.Val190Leu) detected in the current study was de novo, thus supporting its contribution to ns-CP aetiology [46]. A total of 7 out of the 15 tools used for in silico analyses confirmed that this novel substitution may lead to disturbances in protein structure, and the gene had a positive Z score of 1.8, indicating *NHS* intolerance to missense variants. However, since the other bioinformatic programmes used in the study predicted the variant to be neutral, a functional study should be conducted to validate these results.

Missense variant c.1997A>G (p.Tyr666Cys) was also detected in the *NOTCH2* gene, encoding one of the two primary receptors for the NOTCH conservative signalling pathway involved in craniofacial development [47]. After binding to their ligands (Jagged1, Jagged2 or Delta1), the NOTCH1 and NOTCH2 receptors form a transcription complex to regulate gene expression, including *IRF6* [48,49]. The *NOTCH2* gene is highly expressed in the mesenchymal cells within the junction regions of facial primordia during lip and primary palate development in murine models [50]. Moreover, mice without the Jagged2 ligand for the NOTCH2 receptor have a cleft palate [51]. Interestingly, the amino acid substitution p.Tyr666Cys identified in this present study is located within the NOTCH extracellular domain (NECD) composed of the repeating EGF-like motif, which is a ligand-binding site [47], suggesting that p.Tyr666Cys might have a deleterious impact on protein interactions. Moreover, most in silico tools used for pathogenicity predictions confirmed the damaging influence of this substitution on protein. It is worth noting that pathogenic variants within the NECD domain cause Alagille syndrome 2 (OMIM #610205), in which facial dysmorphism is a component of the phenotype [52,53]. To our knowledge, this *NOTCH2* variant is the first reported damaging alternation of this gene detected in the ns-CP patient.

While rare and common variants in T-box family genes, including *TBX1* (OMIM * 602054), *TBX10* (OMIM * 604648) and *TBX22* (OMIM * 300307), have previously been implicated in both syndromic and isolated orofacial clefts [24,26,54,55,56,57,58], the *TBX18* variants have not yet been linked with the risk of these craniofacial anomalies. However, Landson et al. identified a likely damaging copy number variant (CNV) overlapping the *TBX18* gene in a patient with an isolated orofacial cleft [59]. The *TBX18* gene is a member of the family encoding conserved transcriptional factors highly expressed in craniofacial mesenchymal regions during embryogenesis [60]. The *TBX18* variant identified in this current study might be the first reported pathogenic alternation influencing the risk of ns-CP.

Five likely pathogenic variants detected in our ns-CP patients were nucleotide alternations of already-known cleft genes. Interestingly, the de novo variant c.224A>G (p.Asp75Gly) was identified in *IRF6*, which is the major candidate gene engaged in the pathomechanism underlying ns-OFCs. Its crucial role in face development was previously confirmed, and a number of common and rare variants have been described in ns-OFCs patients [5,61]. The list of known candidate genes carrying likely pathogenic variants detected in this current study also included *ARHGAP29*, *FLNB*, *LRP6* and *TP63* [24,26,62,63,64].

Three rare nucleotide variants with strong pathogenicity predictions were detected in genes implicated in the WNT signalling pathway, which is crucial for proper craniofacial development [65]. Among them was the missense variant in *DLG1* encoding essential guanylate kinase, which is involved in proliferative and adhesive processes and the regulation of cell polarity [66,67]. Decreased *DLG1* expression results in impaired functioning of the WNT/β-catenin signalling pathway, leading to craniofacial defects [68,69]. *DLG1* null mice display delayed embryonic development, cleft palate, mandibular hypertrophy, neural tube abnormalities, cardiovascular system disorders, and a lethal respiratory anomaly [70,71]. The *DLG1* p.Glu529Asp substitution disrupts the protein, as evidenced by most in silico prediction algorithms. To our knowledge, the *DLG1* variant described in this study is the first reported damaging alternation of this gene detected in the ns-CP patient. Interestingly, a GWAS conducted in the Polish population has established that common variants located in the *DLG1* locus are significantly associated with the risk of ns-CL/P [41]. Additionally, Pengelly et al. described a rare heterozygous non-frameshift *DLG1* deletion in the ns-CLP family [29].

Other findings supporting the critical role of the WNT signalling pathway in developing craniofacial anomalies are rare, likely pathogenic variants identified in *LRP6* encoding co-receptor of the canonical WNT/β-catenin signalling pathway [72,73] and *WNT5B* encoding signalling ligand implicated in both β-catenin-dependent and β-catenin-independent WNT pathways. Functional studies on animal models have demonstrated that *LRP6* and *WNT5B* knockouts lead to a cleft palate or impaired palatogenesis and short palate, respectively [74,75]. Previous studies have reported the significant association of *LRP6* pathogenic variants with congenital neural tube defects [76] and congenital tooth agenesis [77]. The latter frequently occurs with craniofacial abnormalities, suggesting a common genetic background for these defects [78]. Moreover, mice with *LRP6* deletion exhibit, besides a cleft palate, decreased expression of *MSX1* and *MSX2* [75], well-known candidate genes for both ns-OFC and tooth agenesis [78].

The recent large-scale analyses have shown that the nucleotide variants significantly associated with ns-CP were located mainly within intergenic, deep intronic regions of the genome [17,18,19]; therefore, only analysing the coding sequences of the selected genes is the main limitation of our study. Another study weakness is that the applied tools could not screen for CNVs or larger deletions/insertions, which are essential genetic components contributing to ns-CP [79,80,81]. The small number of patients recruited and the lack of parental DNA samples might also be a further limitation. In addition, rigorous variant filtering and strict prioritisation strategy might lead to missing true causative variants.

## 5. Conclusions

This current study provides further insights into the genetic triggers that may affect the development of the non-syndromic cleft palate and suggests new candidate genes for this craniofacial anomaly.

Moreover, most of the identified likely pathogenic variants were located in genes previously implicated in ns-OFCs; these results confirm the importance of investigating the already-known candidate genes and support the hypothesis that some genes carrying variants associated with syndromic forms of orofacial clefts may also be involved in ns-CP aetiology.

## Figures and Tables

**Table 1:** Characteristics of the patient group.

	All Patients (n = 38)	
	N	%
Gender distribution		
Males	17	44.74
Females	21	55.26
Cleft type		
Cleft of the soft palate	3	7.89
Cleft of the hard and soft palate	35	92.11
Associated anomalies		
Dental anomalies ^1^	8	21.05
Positive family history		
Extended family	4	10.53

^1^ For 21 patients, panoramic radiographs were not available for analysis.

**Table 2:** Nucleotide variants identified in patients with ns-CP.

						Variant Effect		gnomAD	Genotyping Results ^c^		
Patient	Gender	Type of Cleft	Family History ^a^	Gene	Rs Number	DNA Change	Protein Change	Frequency ^b^	Patient	Father	Mother
*De novo* Variants											
CP_1	M	Cleft of the hard and soft palate	NO	*IRF6*	novel	c.224A>G	p.Asp75Gly	not found	HET	wt	wt
CP_2	F	Cleft of the hard and soft palate	YES—ex. fam.	*NHS*	novel	c.568G>C	p.Val190Leu	not found	HET	wt	wt
Variants Inherited from Unaffected Parent											
CP_3	F	Cleft of the hard and soft palate	NO	*COL17A1*	novel	c.2435-1G>A		not found	HET	wt	HET
CP_4	F	Cleft of the hard and soft palate	YES—ex. fam.	*DLG1*	rs139789027	c.1586G>C	p.Glu562Asp	2.95 × 10^−5^	HET	wt	HET
CP_5	F	Cleft of the hard and soft palate	NO	*FLNB*	novel	c.3605A>G	p.Tyr1202Cys	not found	HET	no DNA	HET
CP_6	F	Cleft of the hard and soft palate	NO	*LRP6*	rs769446193	c.481C>A	p.Pro161Thr	not found	HET	no DNA	HET
CP_7	F	Cleft of the hard and soft palate	NO	*NOTCH2*	novel	c.1997A>G	p.Tyr666Cys	not found	HET	HET	wt
CP_8	M	Cleft of the hard and soft palate	NO	*TP63*	novel	c.353A>T	p.Asn118Ile	not found	HET	wt	HET
CP_9	F	Cleft of the hard and soft palate	NO	*VAX1*	novel	c.400G>A	p.Ala134Thr	not found	HET	wt	HET
CP_10	M	Cleft of the hard and soft palate	NO	*WNT5B*	rs529807731	c.716G>T	p.Arg239Leu	0.000	HET	HET	wt
Variants with Unknown Inheritance											
CP_11	M	Cleft of the hard and soft palate	NO	*ARHGAP29*	rs867470445	c.1706G>A	p.Arg569Gln	1.47 × 10^−5^	HET	no DNA	wt
CP_12	F	Cleft of the soft palate	NO	*TBX18*	novel	c.674A>T	p.His225Leu	not found	HET	no DNA	wt

^a^ ex. fam., extended family. ^b^ The Genome Aggregation Database v.3.1.1 [gnomAD, genome sequencing data, population: European (non-Finish)] https://gnomad.broadinstitute.org/ (accessed on 1 March 2022). ^c^ Sanger sequencing; HET, heterozygote; wt, wild-type.

**Table 3:** Pathogenicity scores of variants detected in ns-CP patients.

GENE			VARIANT		*IN SILICO* PATHOGENICITY PREDICTION ^c^																	ADA	CADD
Name	pLi ^a^	Z score ^b^	Alternation	Type	1	2	3	4	5	6	7	8	9	10	11	12	13	14	15	16	17	Score ^d^	Score ^e^
*De novo* Variants																							
*IRF6*	1.000	2.774	c.224A>G; p.Asp75Gly	Missense	Dc	M	D	D	D	D	D	P	P	D	D	D	P	P	D	D	D		28.5
*NHS*	1.000	1.822	c.568G>C; p.Val190Leu	Missense	Dc	N	T	D	D	D	T			T, D	T, D	N	B	B	D	T	T		24.6
Variants Inherited from Unaffected Parent																							
*COL17A1*	0.000	−0.748	c.2435-1G>A	Splicing	D			D				P	P									0.99	33.0
*DLG1*	0.994	1.496	c.1586G>C; p.Glu562Asp	Missense	Dc	M	D	D	N	D	T	B	P	D	D	N	B	B	T	D	D		23.6
*FLNB*	0.000	2.141	c.3605A>G; p.Tyr1202Cys	Missense	Dc	M	T	D	D	D	D	P	P	D	D	D	P	P	D	D	D		29.2
*LRP6*	0.697	2.754	c.481C>A; p.Pro161Thr	Missense	Dc	H	D	D	D	D	D	P	P	D	D	D	P	P	D	D	D		26.0
*NOTCH2*	1.000	3.502	c.1997A>G; p.Tyr666Cys	Missense	Dc	H	D	D	D	U	D	P	P	D	D	D	P	P	T	D	D		26.1
*TP63*	0.997	2.208	c.353A>T; p.Asn118Ile	Missense	Dc	L	T, D	D	D	D	D	P	P	D	D	D	P	P	D	D	D		27.6
*VAX1*	0.771	0.869	c.400G>A; p.Ala134Thr	Missense	Dc	H	D	D	D	D	D	P	P	D	D	D	P	P	D	D	D		27.6
*WNT5B*	0.589	1.788	c.716G>T; p.Arg239Leu	Missense	Dc	H	T	D	D	D	D	P	P	D	D	D	B	P	D	D	D		32.0
Variants with Unknown Inheritance																							
*ARHGAP29*	1.000	1.211	c.1706G>A; p.Arg569Gln	Missense	Dc	M	T	D	D	N	T	P	P	D	D	D	B	B	D	T	T		28.4
*TBX18*	0.999	0.099	c.674A>T; p.His225Leu	Missense	Dc	H	D	D	D	D	D	P	P	D	D	D	P	P	D	D	D		31.0

^a^ pLi, the probability of being loss-of-function (LoF) intolerant; pLi ≥ 0.9 indicates that gene is LoF intolerant—The Genome Aggregation Database v.2.1.1. https://gnomad.broadinstitute.org/ (accessed on1 March 2022 ). ^b^ Z score indicates a gene’s intolerance to missense variants; positive Z scores indicate increased constraint (intolerance to variation)—The Genome Aggregation Database v.2.1.1. ^c^ Pathogenicity of missense variants was predicted using 17 in silico tools (1—MutationTaster, 2—MutationAssessor, 3—FATHMM, 4—FATHMM-MKL, 5—FATHMM-XF, 6—LRT, 7—DEOGEN2, 8—EIGEN, 9—EIGEN PC, 10—SIFT, 11—SIFT4G, 12—PROVEAN, 13—MVP, 14—REVEL, 15—PrimateAI, 16—MetaSVM and 17—MetaLR). B, Benign; D, Damaging/Deleterious; Dc, Disease causing; H, High; L, Low; M, Medium; N, Neutral; P, Pathogenic; T, Tolerated; U, Uncertain. ^d^ Adaptive boosting (ADA) score for variants predicted to affect splicing; the ADA score close to 1 indicates a high probability of being splice-affecting variant—dbNSFP database. ^e^ CADD—Combined Annotation Dependent Depletion version 1.6. https://cadd.gs.washington.edu/ (accessed on 1 March 2022).

## Data Availability

The de-identified datasets generated through this study can be provided by the corresponding author upon request.

## Supplementary Materials

The following supporting information can be downloaded at: https://www.mdpi.com/article/10.3390/jcm12052051/s1, Table S1: The NGS-targeted multi-gene panel for ns-CP—characteristics of genes; Table S2: The NGS-targeted multi-gene panel for ns-CP—phenotype of knockout mouse models; Table S3: Characteristics of likely pathogenic variants fulfilling the project’s strict selection criteria, which were identified in ns-CP patients; Table S4: Characteristic of all likely pathogenic variants, including variants not fulfilling the project’s strict selection criteria, which were identified in ns-CP patients; Figure S1: Flow chart of processing next-generation sequencing data; Figure S2: Detection of a novel *IRF6* variant in a patient with non-syndromic cleft of the hard and soft palate; Figure S3: Detection of a novel *NHS* variant in a patient with non-syndromic cleft of the hard and soft palate; Figure S4: Detection of a novel *COL17A1* variant in a patient with non-syndromic cleft of the hard and soft palate; Figure S5: Detection of a known *DLG1* variant in a patient with non-syndromic cleft of the hard and soft palate; Figure S6: Detection of a novel *FLNB* variant in a patient with non-syndromic cleft of the hard and soft palate; Figure S7: Detection of a known *LRP6* variant in a patient with non-syndromic cleft of the hard and soft palate; Figure S8: Detection of a novel *NOTCH2* variant in a patient with non-syndromic cleft of the hard and soft palate; Figure S9: Detection of a novel *TP63* variant in a patient with non-syndromic cleft of the hard and soft palate; Figure S10: Detection of a novel *VAX1* variant in a patient with non-syndromic cleft of the hard and soft palate; Figure S11: Detection of a known *WNT5B* variant in a patient with non-syndromic cleft of the hard and soft palate; Figure S12: Detection of a known *ARHGAP29* variant in a patient with non-syndromic cleft of the hard and soft palate; Figure S13: Detection of a novel *TBX18* variant in a patient with cleft of the soft palate.
