# Supplementary material for: Identification of Novel Risk Variants of Non-Syndromic Cleft Palate by Targeted Gene Panel Sequencing
Source: J Clin Med. 2023 Mar 4;12(5):2051. doi: 10.3390/jcm12052051 (PMC10004578; doi:10.3390/jcm12052051)
Supplement: Supplementary file 1 [file jcm-12-02051-s001.zip › Supplementary Figures 1-13.pdf]

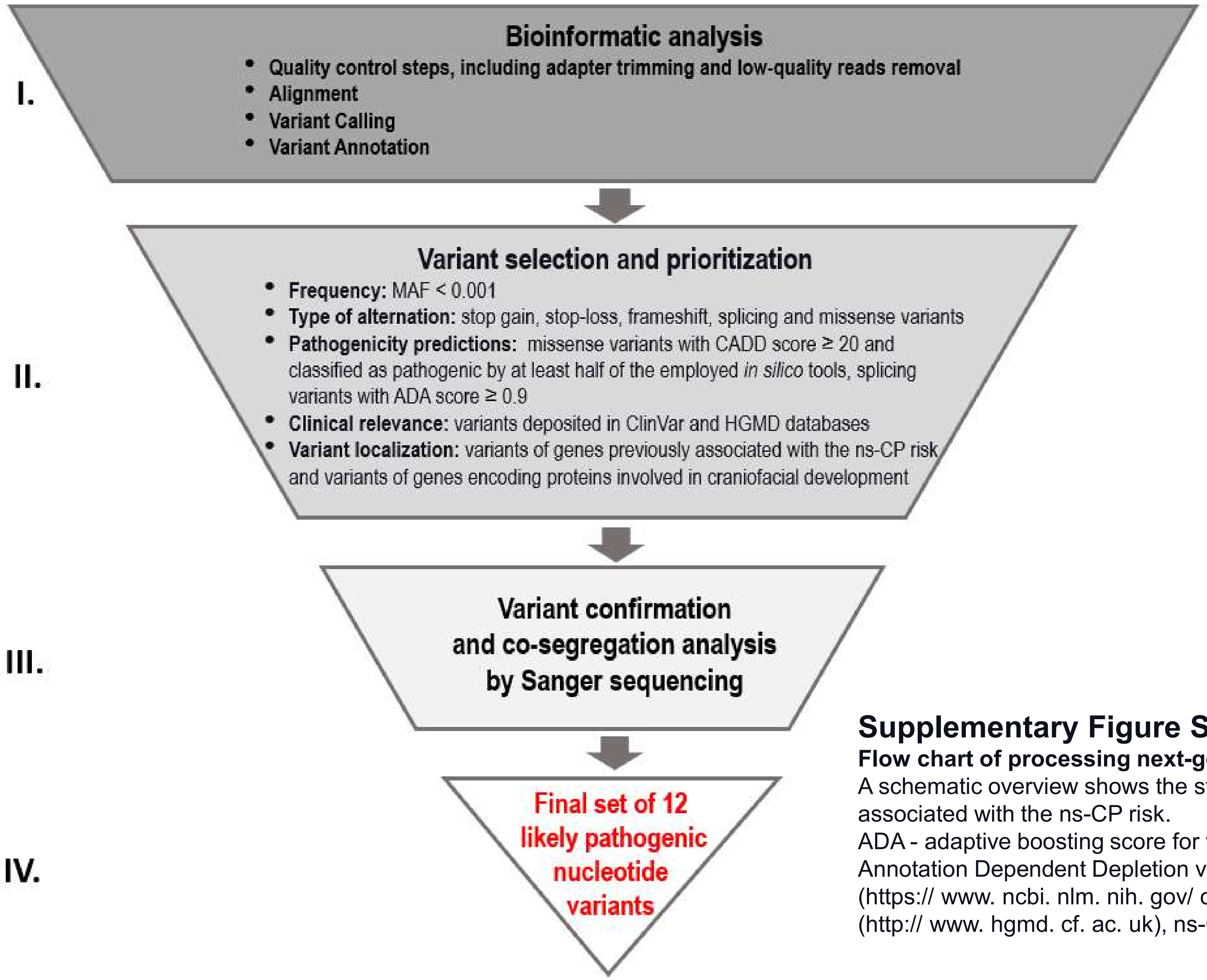

Supplementary Figure S1

Flow chart of processing next-generation sequencing data.

A schematic overview shows the strategy used to select likely pathogenic variants associated with the ns-CP risk.

ADA - adaptive boosting score for variants predicted to affect splicing, CADD - Combined Annotation Dependent Depletion version 1.6. (https://cadd.gs.washington.edu/), ClinVar (https:// www. ncbi. nlm. nih. gov/ clinv ar/), HGMD - Human Gene Mutation Database (http:// www. hgmd. cf. ac. uk), ns-CP – non-sydromic cleft palate.

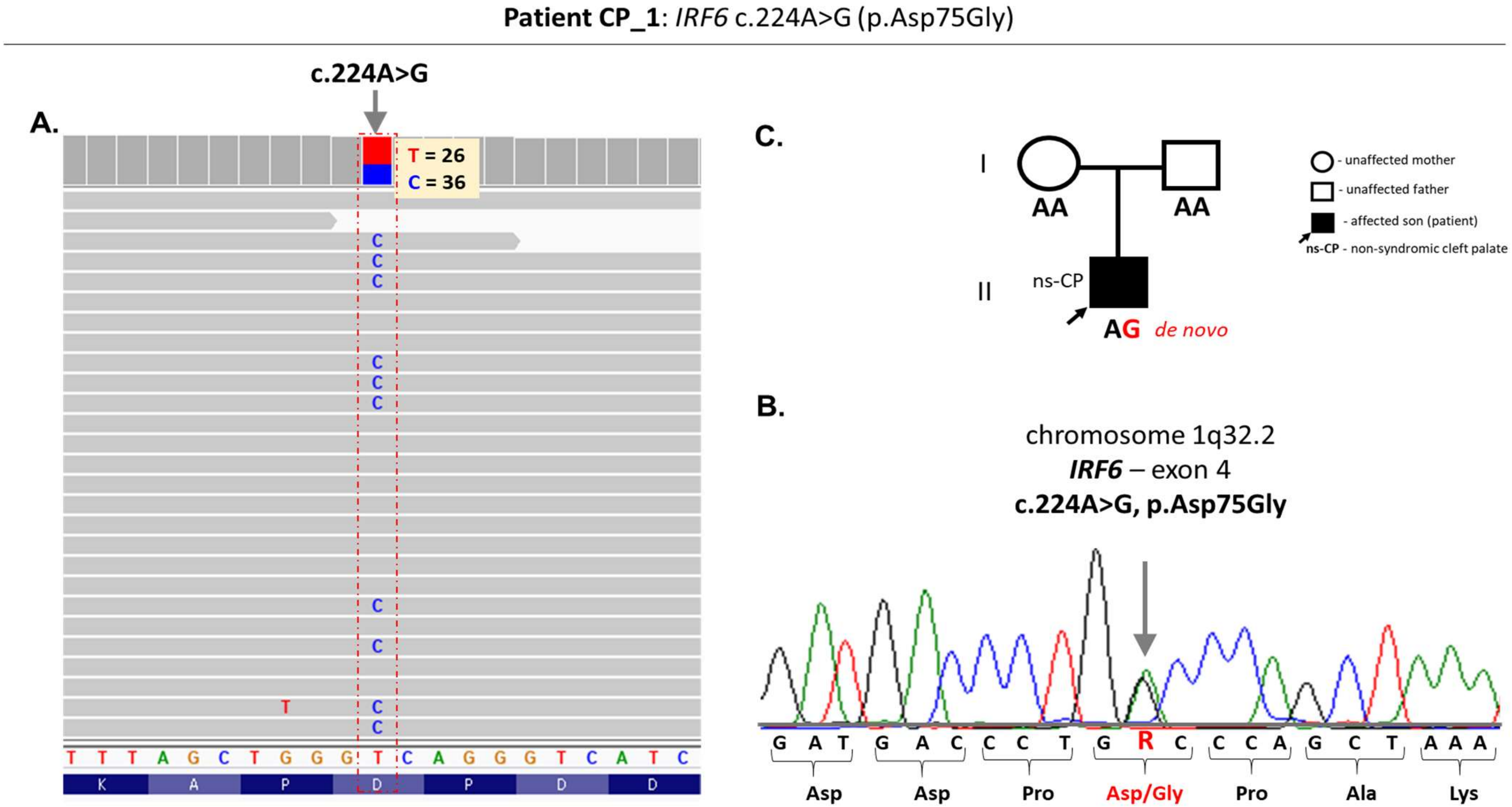

Supplementary Figure S2

Detection of a novel *IRF6* variant in a patient with non-syndromic cleft of the hard and soft palate

(A) the targeted next-generation sequencing identified heterozygous c.224A>G variant in exon 4 of the *IRF6* gene, leading to p.Asp75Gly substitution, (B) the presence of this transition was confirmed by Sanger sequencing, (C) the segregation analysis revealed that the variant arose *de novo.*

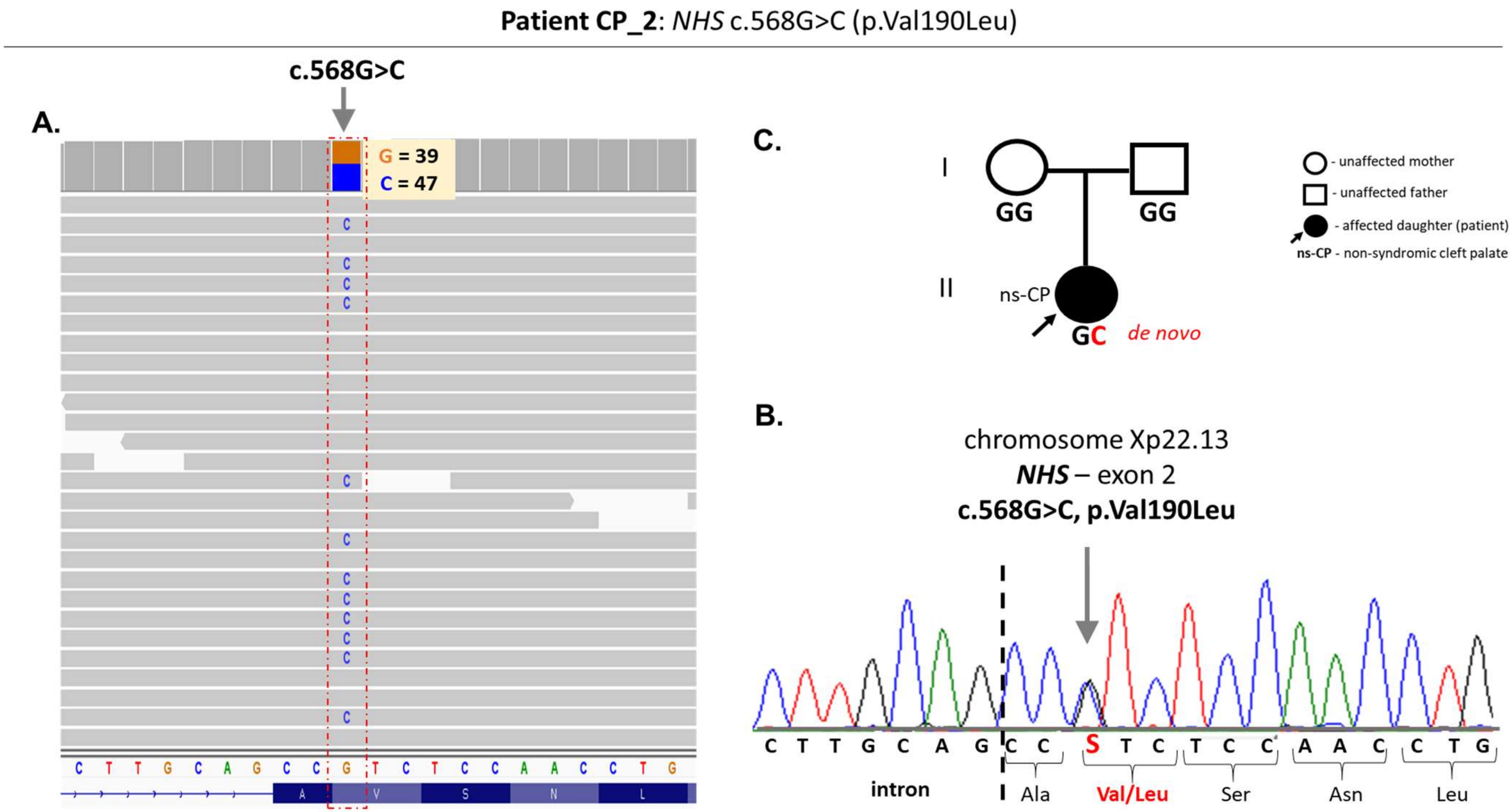

Supplementary Figure S3

Detection of a novel *NHS* variant in a patient with non-syndromic cleft of the hard and soft palate

(A) the targeted next-generation sequencing identified heterozygous c.568G>C variant in exon 2 of the *NHS* gene, leading to p.Val190Leu substitution, (B) the presence of this transversion was confirmed by Sanger sequencing, (C) segregation analysis revealed that the variant arose *denovo*.

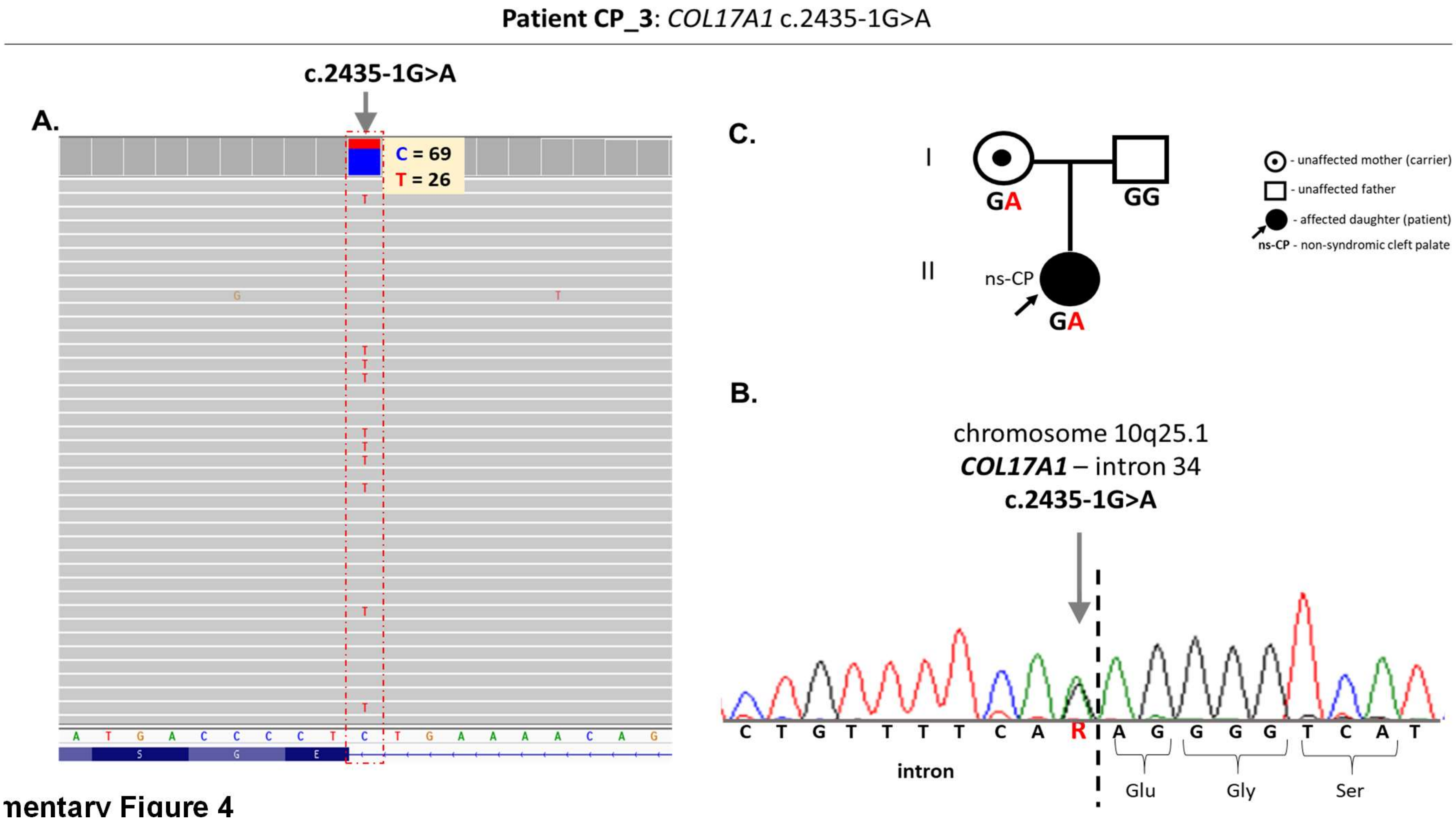

Supplementary Figure S4

Detection of a novel *COL17A1* variant in a patient with non-syndromic cleft of the hard and soft palate

(A) the targeted next-generation sequencing identified heterozygous c.2435-1G>A splicing variant in intron 34 of the *COL17A1* gene, (B) the presence of this transition was confirmed by Sanger sequencing, (C) the segregation analysis revealed that the patient inherited the c.2435-1G>A nucleotide variant from unaffected mother.

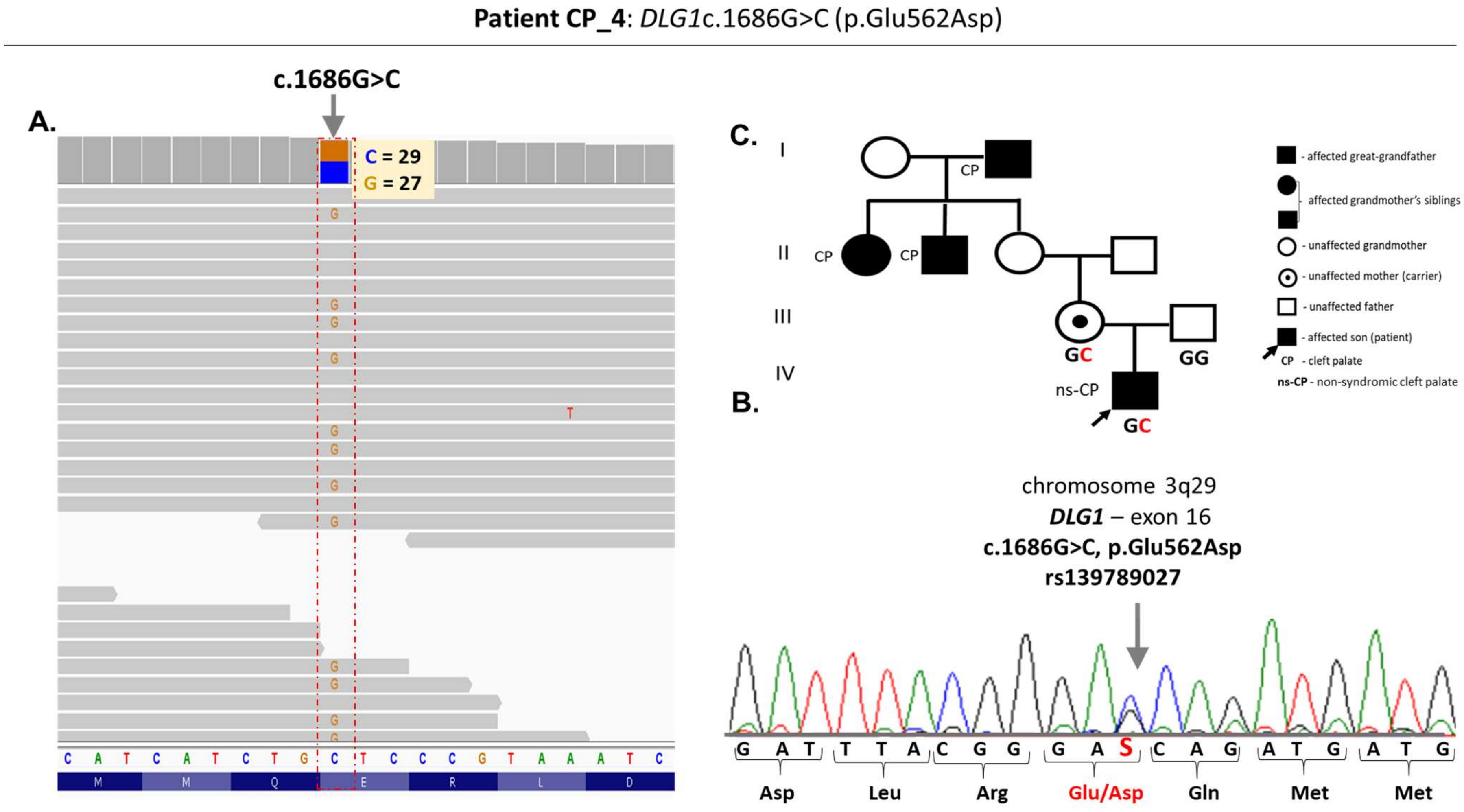

Supplementary Figure S5

Detection of a known *DLG1* variant in a patient with non-syndromic cleft of the hard and soft palate

(A) the targeted next-generation sequencing identified heterozygous c.1686G>C (rs139789027) variant in exon 16 of the *DLG1* gene, leading to p.Glu562Asp substitution, (B) the presence of this transversion was confirmed by Sanger sequencing, (C) segregation analysis revealed that the patient inherited the c.1686G>C nucleotide variant from unaffected mother; cleft palate was also observed in the patient's maternal grandmother siblings and great-grandfather.

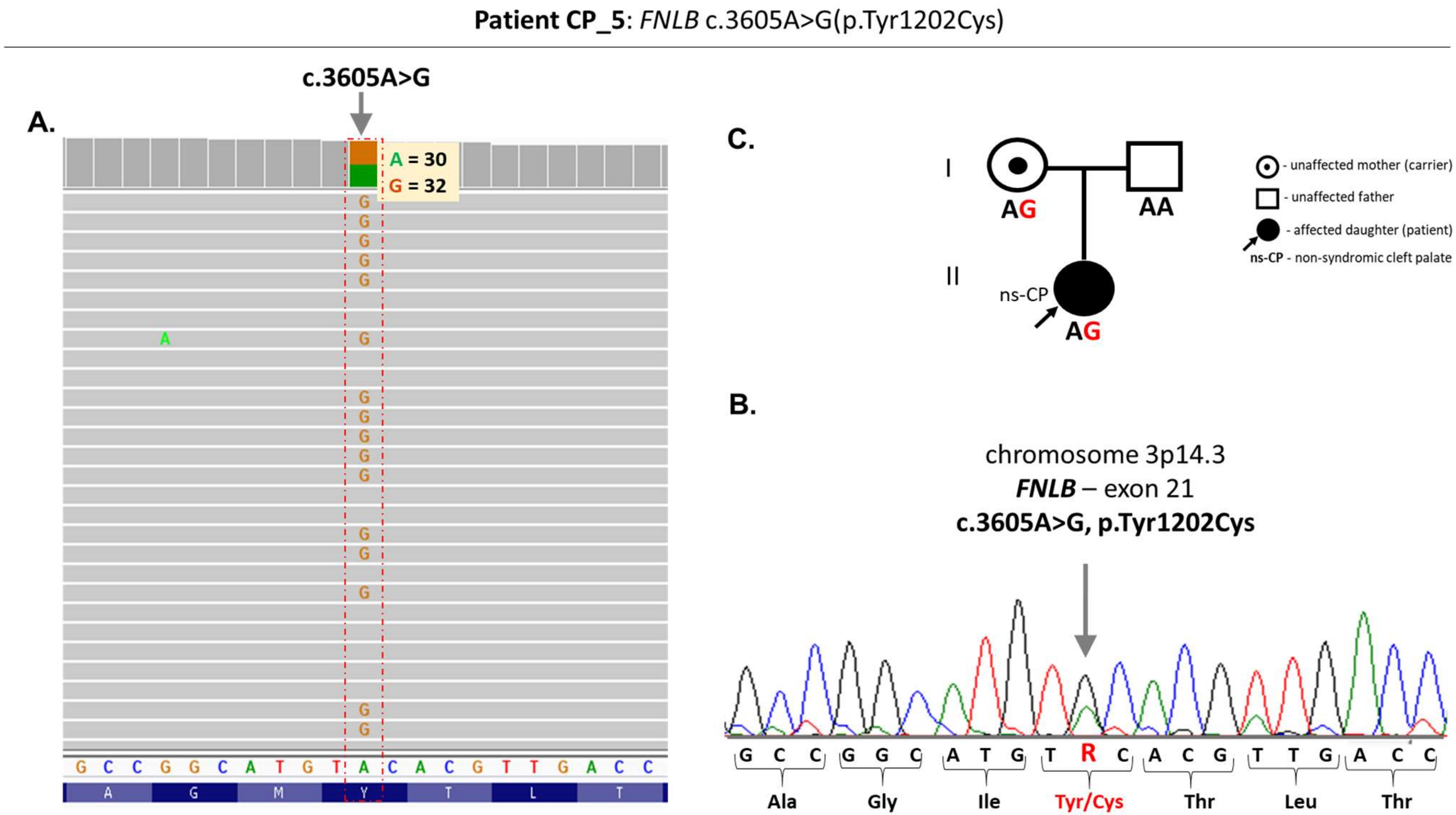

Supplementary Figure S6

Detection of a novel *FLNB* variant in a patient with non-syndromic cleft of the hard and soft palate

(A) the targeted next-generation sequencing identified heterozygous c.3605A>G variant in exon 21 of the *FLNB* gene, leading to p.Tyr1202Cys substitution, (B) the presence of this transition was confirmed by Sanger sequencing, (C) the segregation analysis revealed that the patient inherited the c.3605A>G nucleotide variant from unaffected mother.

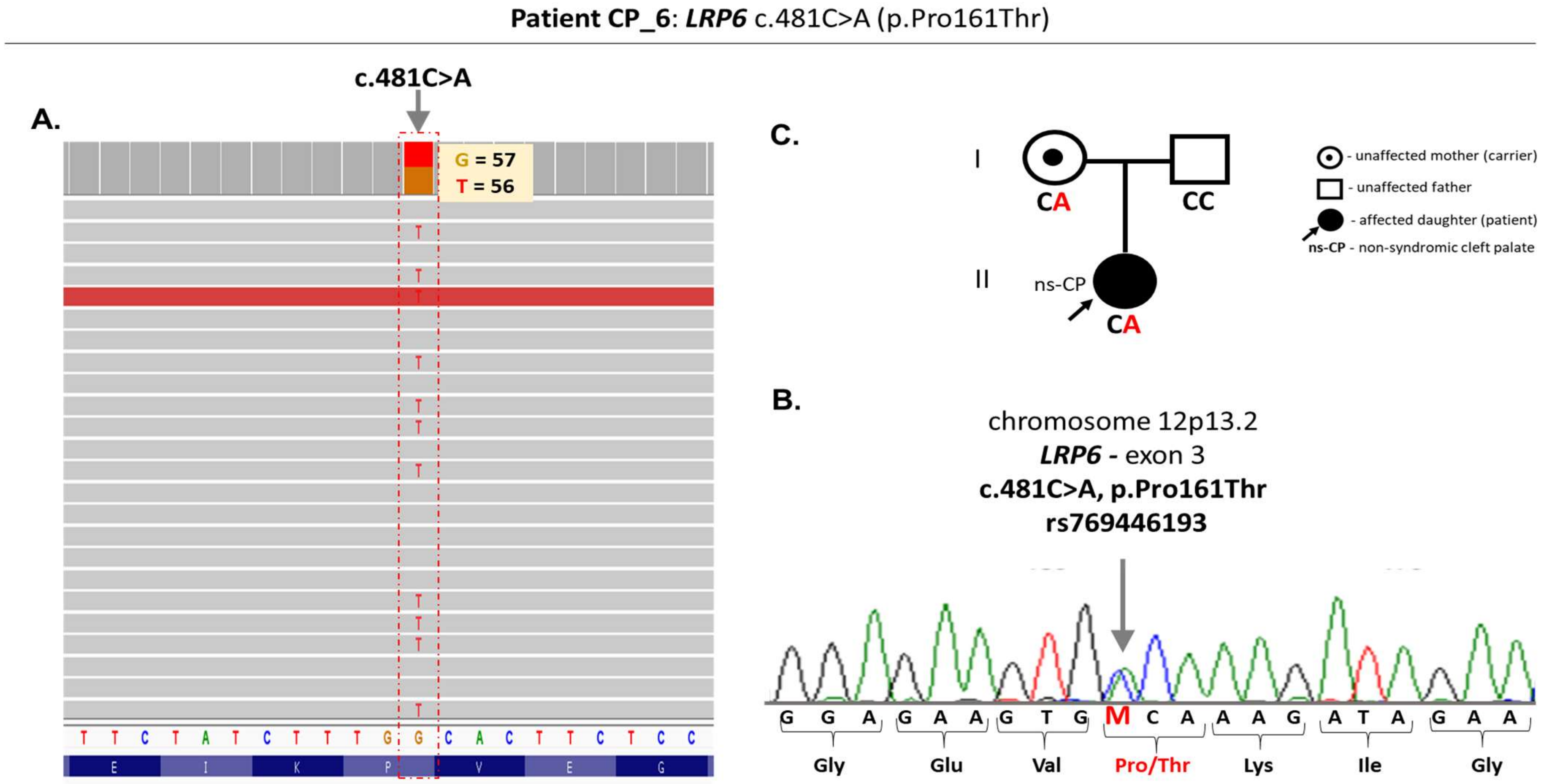

Supplementary Figure S7

Detection of a known *LRP6* variant in apatient with non-syndromic cleft of the hard and soft palate

(A) the targeted next-generation sequencing identified heterozygous c.481C>A (rs769446193) variant in exon 3 of the *LRP6* gene, leading to p.Pro161Thr substitution, (B) the presence of this transversion was confirmed by Sanger sequencing, (C) the segregation analysis revealed that the patient inherited the c.481C>A nucleotide variant from unaffected mother.

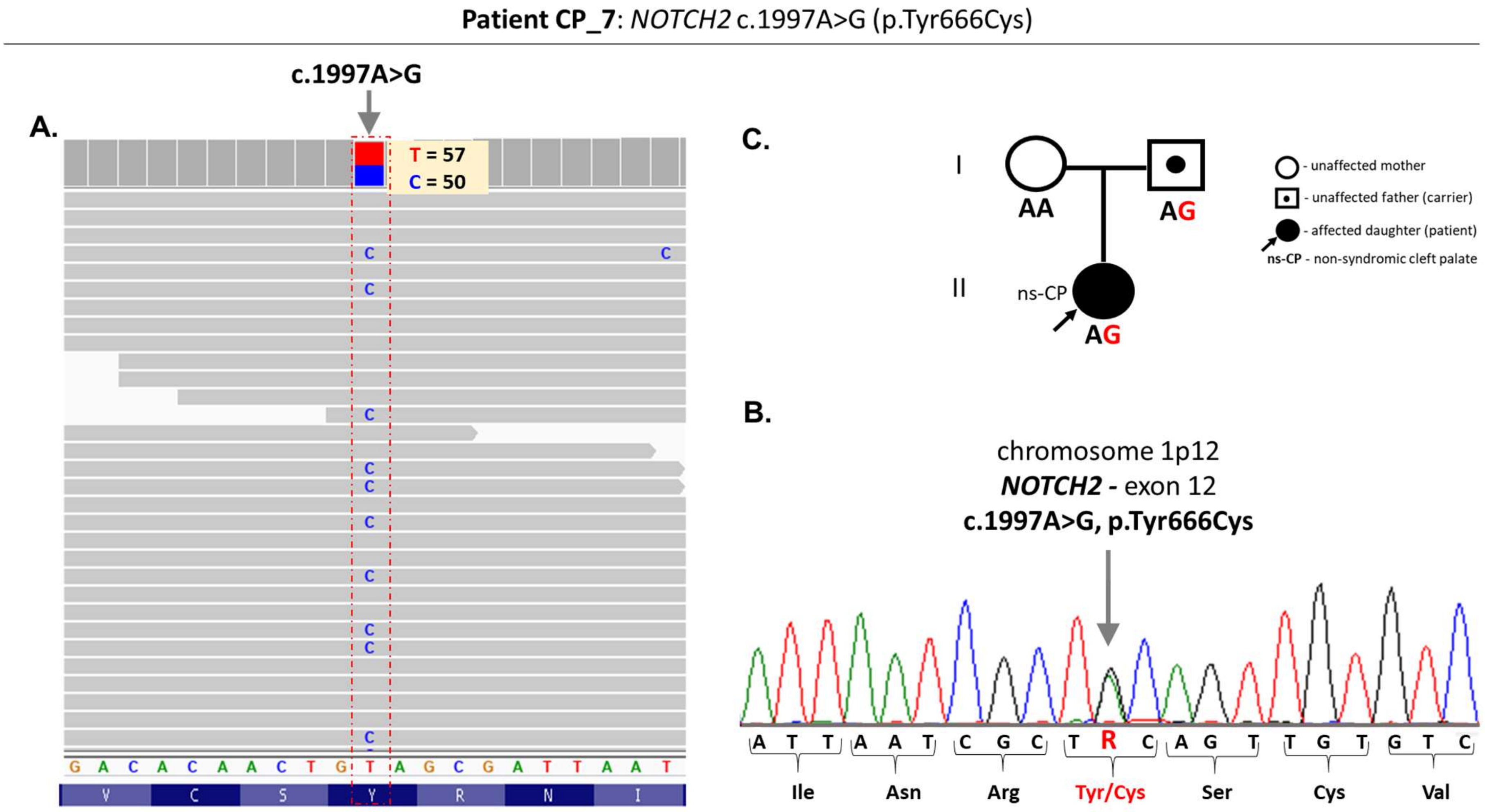

Supplementary Figure S8

Detection of a novel *NOTCH2*variant in a patient with non-syndromic cleft of the hard and soft palate

(A) the targeted next-generation sequencing identified heterozygous c.1997A>G variant in exon 12 of the *NOTCH2* gene, leading p.Tyr666Cys to substitution, (B) the presence of this transition was confirmed by Sanger sequencing, (C) the segregation analysis revealed that the patient inherited the c.1997A>G nucleotide variant from unaffected father.

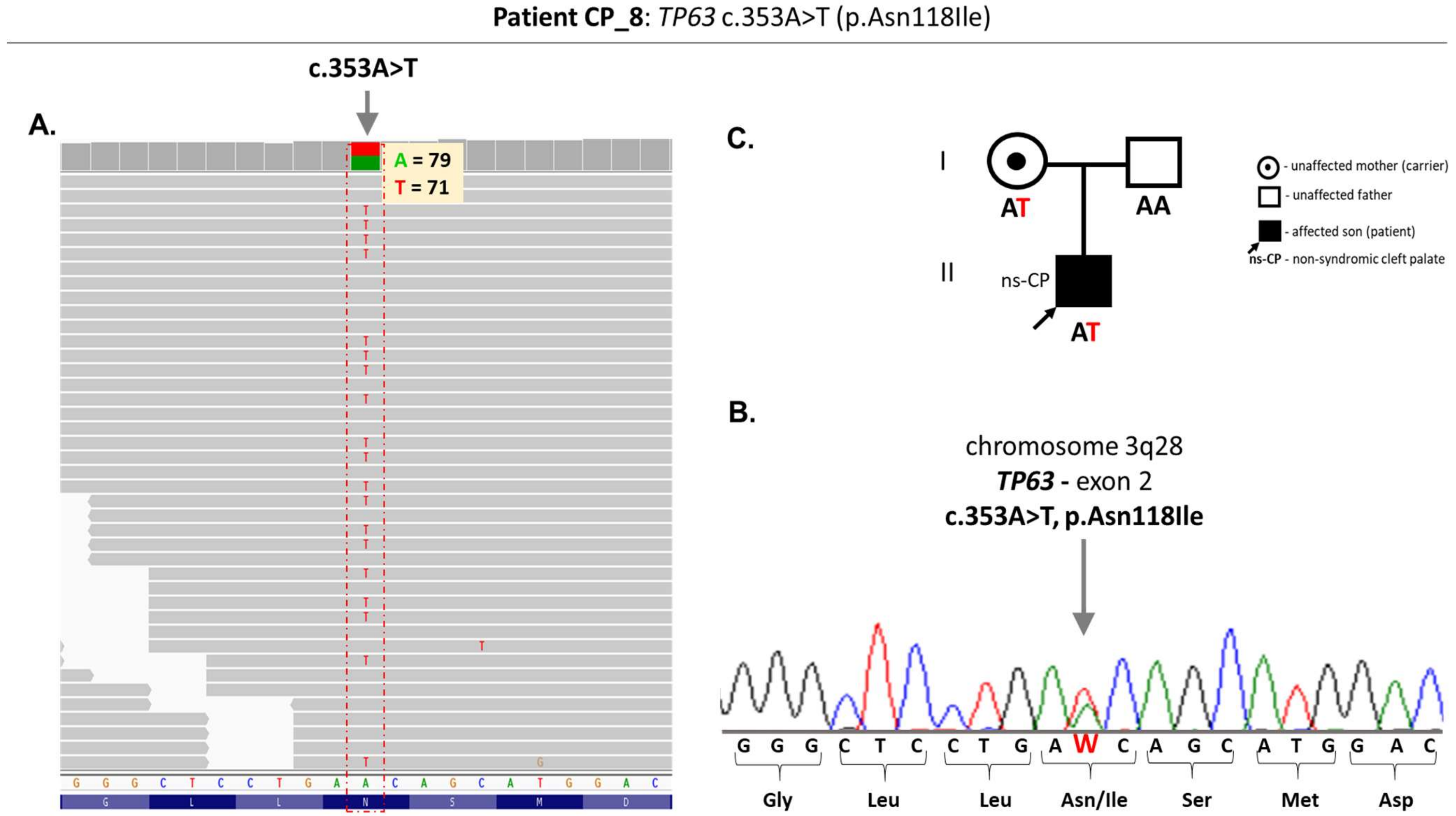

Supplementary Figure S9

Detection of a novel *TP63*variant in a patient with non-syndromic cleft of the hard and soft palate

(A) the targeted next-generation sequencing identified heterozygous c.353A>T variant in exon 2 of the *TP63*gene, leading to p.Asn118Ile substitution, (B) the presence of this transversion was confirmed by Sanger sequencing, (C) segregation analysis revealed that the patient inherited the c.353A>T nucleotide variant from unaffected mother.

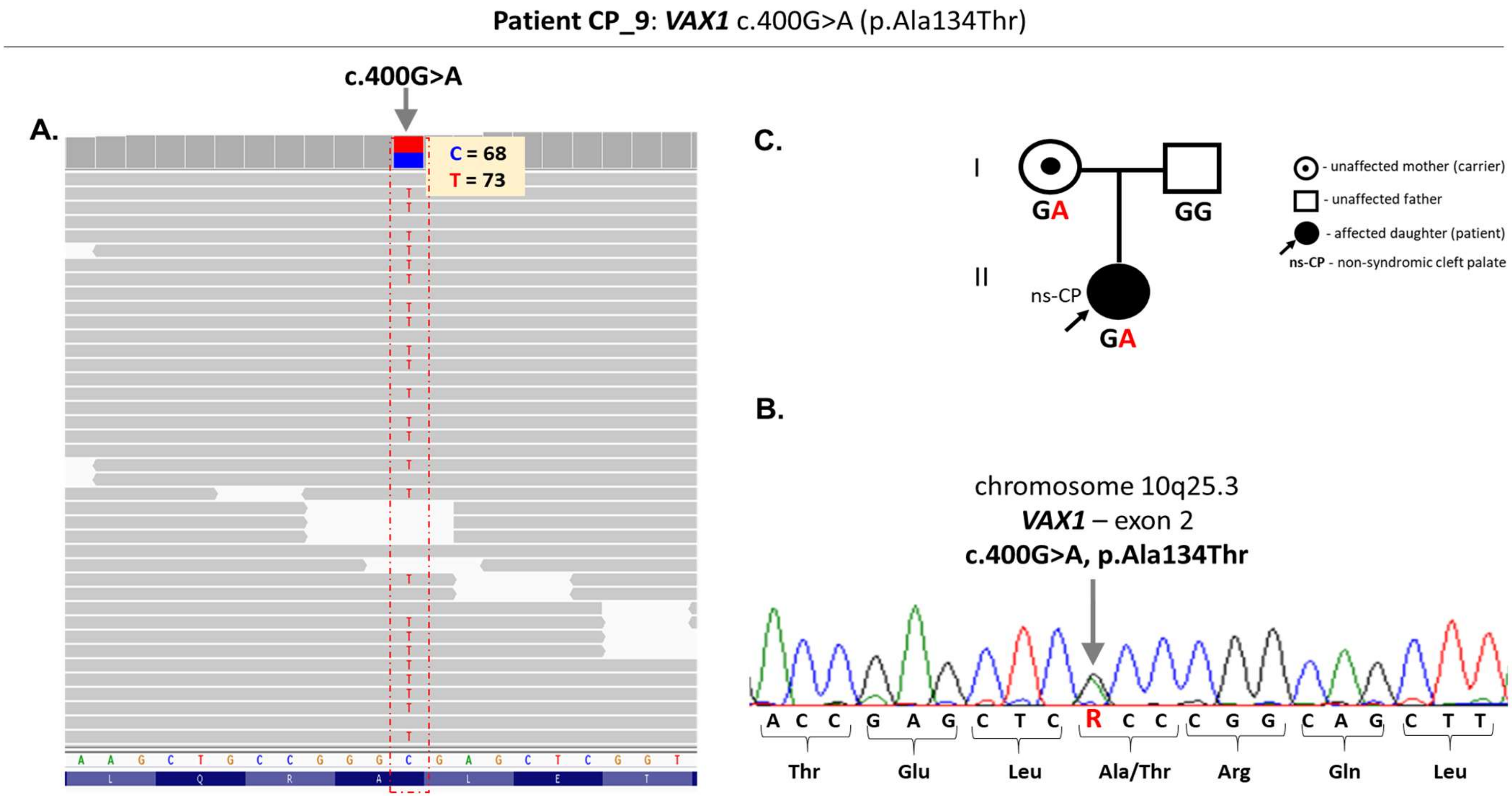

Supplementary Figure S10

Detection of a novel *VAX1* variant in a patient with non-syndromic cleft of the hard and soft palate

(A) the targeted next-generation sequencing identified heterozygous c.400G>A variant in exon 2 of the *VAX1* gene, leading to p.Ala134Thr substitution, (B) the presence of this transition was confirmed by Sanger sequencing, (C) segregation analysis revealed that the patient inherited the c.400G>A nucleotide variant from unaffected mother.

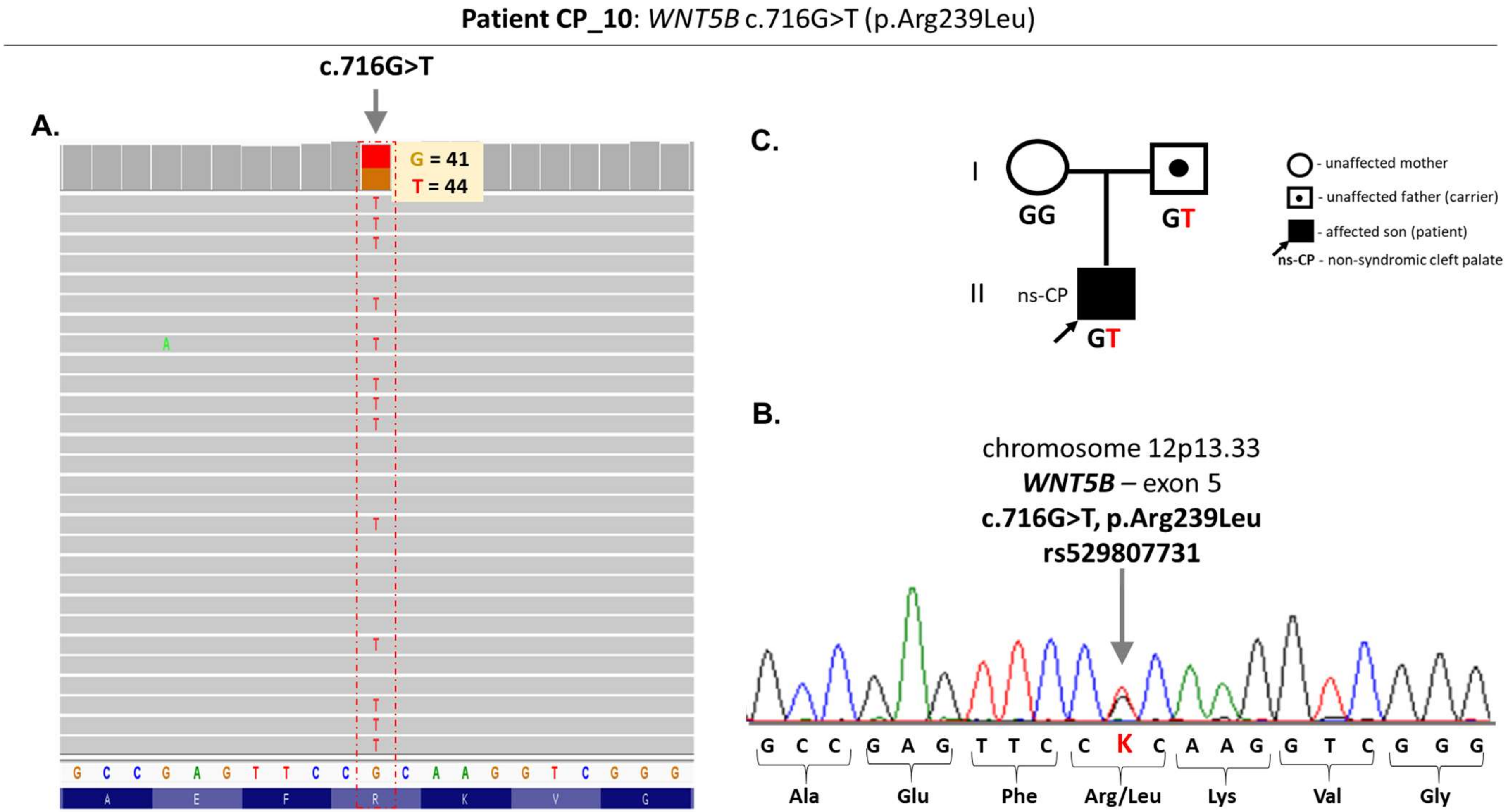

Supplementary Figure S11

Detection of a known *WNT5B* variant in a patient with non-syndromic cleft of the hard and soft palate

(A) the targeted next-generation sequencing identified heterozygous c.716G>T (rs529807731) variant in exon 5 of the *WNT5B* gene, leading to p.Arg239Leu substitution, (B) the presence of this transversion was confirmed by Sanger sequencing, (C) the segregation analysis revealed that the patient inherited the c.716G>T nucleotide variant from unaffected mother.

Patient CP_11: *ARHGAP29* c.1706G>A (p.Arg569Gln)

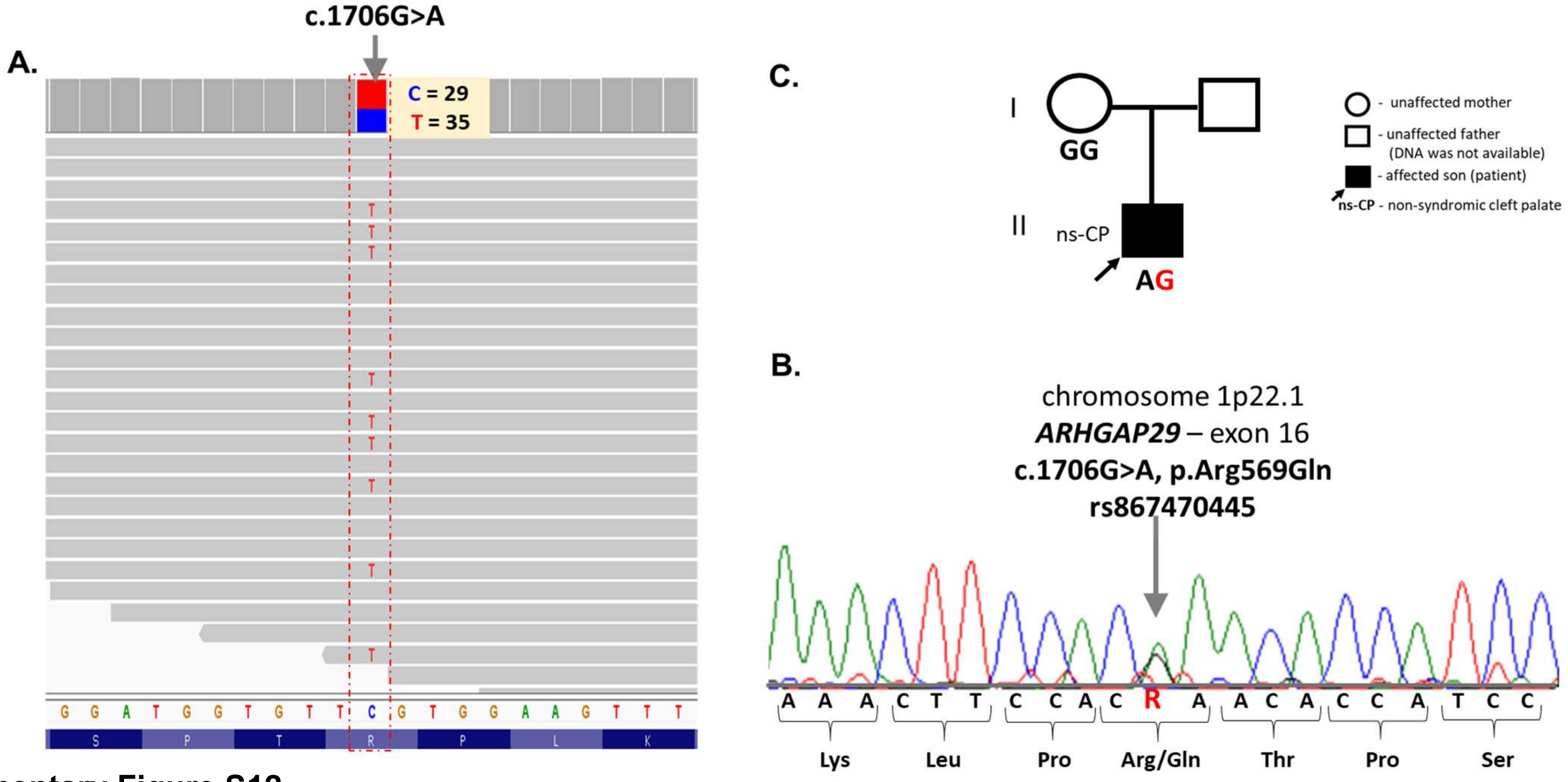

Supplementary Figure S12

Detection of a known *ARHGAP29* variant in a patient with non-syndromic cleft of the hard and soft palate

(A)targeted next-generation sequencing identified heterozygous c.1706G>A (rs867470445) variant in exon 16 of the *ARHGAP29* gene, leading to p.Arg569Gln substitution,(B) the presence of this transition was confirmed by Sanger sequencing, (C) segregation analysis due to the lack of DNA sample from father was impossible.

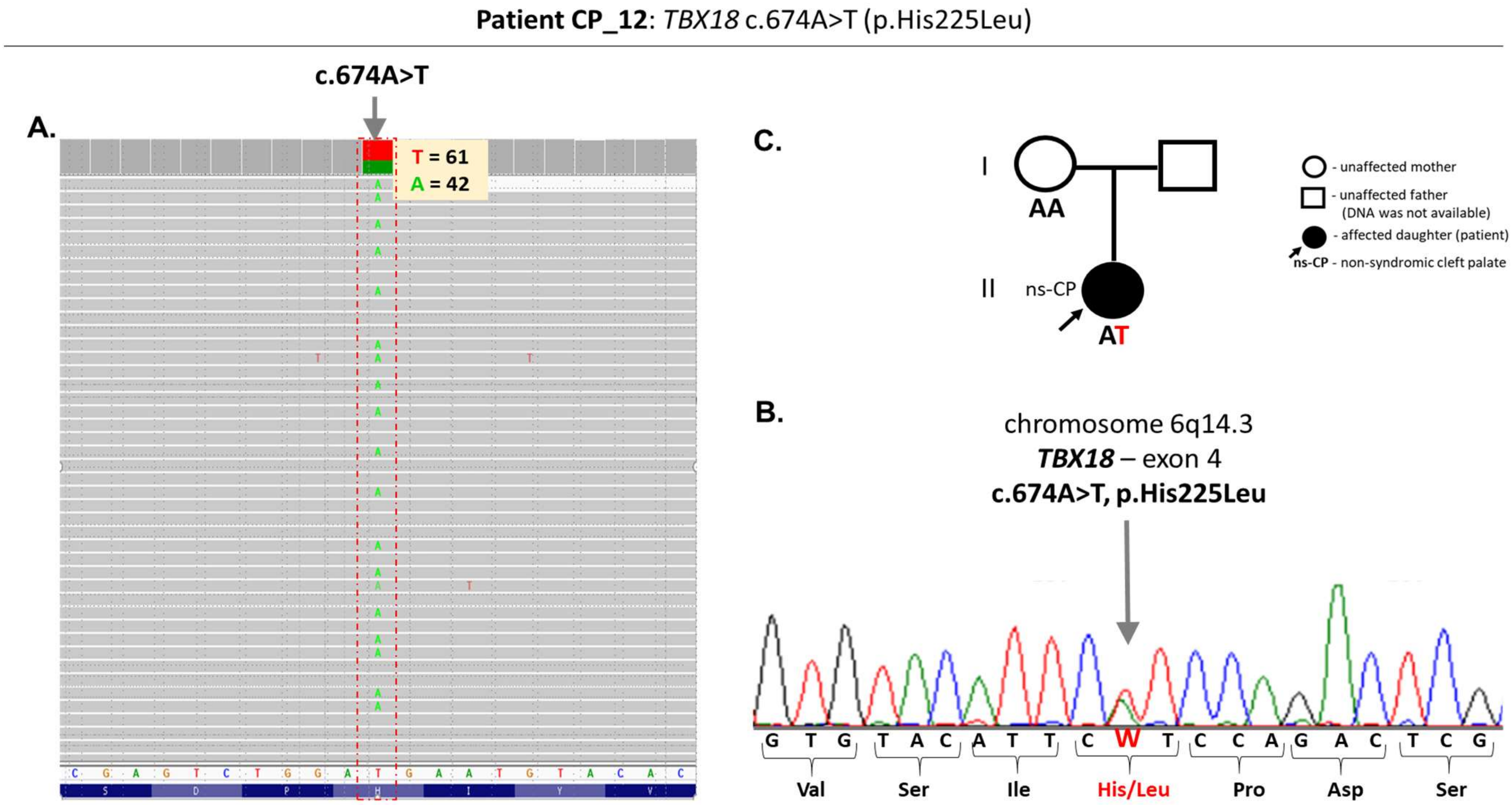

Supplementary Figure S13

Detection of a novel *TBX18* variant in a patient with cleft of soft palate

(A) the targeted next-generation sequencing identified heterozygous c.674A>T variant in exon 4 of the *TBX18* gene, leading to p.His225Leu substitution, (B) presence of this transversion was confirmed by Sanger sequencing, (C) segregation analysis due to the lack of DNA sample from father was impossible.
